# Toward climate resilience in Israel’s healthcare system: decision-makers' perspectives

**DOI:** 10.1186/s13584-025-00729-w

**Published:** 2025-11-24

**Authors:** Irit Lador, Maya Negev, Anat Rosenthal, Stav Shapira

**Affiliations:** 1 School of Public Health, Faculty of Health and Welfare Sciences, University of Haifa, Haifa, Israel; 2 Department of Health Policy and Management, School of Public Health, Faculty of Health Sciences, Ben-Gurion University of the Negev, Beer-Sheva, Israel; 3 School of Public Health, Faculty of Health Sciences, Ben-Gurion University of the Negev, Beer-Sheva, Israel

**Keywords:** Climate adaptation, Climate change, Climate resilience, Health policy, Health systems

## Abstract

**Background:**

Climate change is increasingly recognized as a major global health threat, highlighting the need to enhance climate resilience within health systems. The World Health Organization (WHO) defines key components for climate-resilient health systems, including governance, financing, workforce, and emergency preparedness. Israel is particularly vulnerable to climate change, which is expected to exacerbate the strain on a healthcare system already facing budget cuts, staff shortages, equipment deficits, and security challenges. We aimed to analyze the barriers to and facilitators of climate resilience initiatives in Israel’s healthcare system from the perspective of decision-makers. Understanding the unique contextual factors within Israel’s healthcare system can inform the development of a tailored climate and health framework and shape national policy.

**Methods:**

In this qualitative study, semi-structured interviews were conducted with 25 decision-makers, administrators, and professionals from the Ministry of Health, hospitals, and health maintenance organizations (HMOs). Participants were selected using purposive sampling to ensure diverse representation. Data were analyzed through deductive thematic analysis, guided by the Consolidated Framework for Implementation Research (CFIR).

**Results:**

The absence of a national policy framework, including dedicated funding and binding regulations, emerged as a central barrier to advancing climate resilience within the Israeli healthcare system. Additionally, the study revealed that climate issues are deprioritized due to security and budgetary constraints, coupled with limited awareness of climate risks. Economic incentives were frequently cited as enablers for promoting climate resilience. Despite challenges, the findings highlight the potential for integrating climate resilience into existing emergency preparedness systems.

**Conclusions:**

The study underscores significant gaps in climate resilience within Israel’s healthcare system, particularly the lack of a coordinated, government-led framework for climate adaptation. Although local efforts exist, they remain fragmented and unsustainable without national leadership and funding. Key recommendations include developing a comprehensive national health and climate plan, securing dedicated funding, and increasing awareness/training for healthcare professionals.

**Supplementary Information:**

The online version contains supplementary material available at 10.1186/s13584-025-00729-w.

## Background

Climate change is recognized as the 21 st century’s foremost global health threat [1], contributing to a rise in all-cause mortality and morbidity, heat stress, respiratory illness and allergies, and deteriorating mental health [2]. The most common strategies to address climate change include mitigation (aiming to reduce greenhouse gas emissions) and adaptation (aiming to enhance the capacity of systems and communities to cope with its inevitable consequences) [3]. The healthcare sector is heavily impacted by, but also contributes significantly to, climate change, accounting for approximately 4–5% of global greenhouse gas emissions through energy use, transportation, and the full life cycle of medical products [4, 5]. Such challenges underscore the critical need for strengthening climate resilience: the capacity of healthcare systems to anticipate, prepare for, respond to, and recover from climate-related risks while actively promoting low-carbon sustainability [6].

Climate-related risks result from the dynamic interaction between climate-related hazards (e.g., heatwaves, floods, and droughts) and the affected human or ecological system’s vulnerability to these hazards [7;95;96]. Climate change negatively impacts healthcare facilities, especially through increased patient admissions [8], and exacerbates healthcare access challenges, disrupts system continuity, and magnifies health inequities [9–12].

**Fig. 1:**
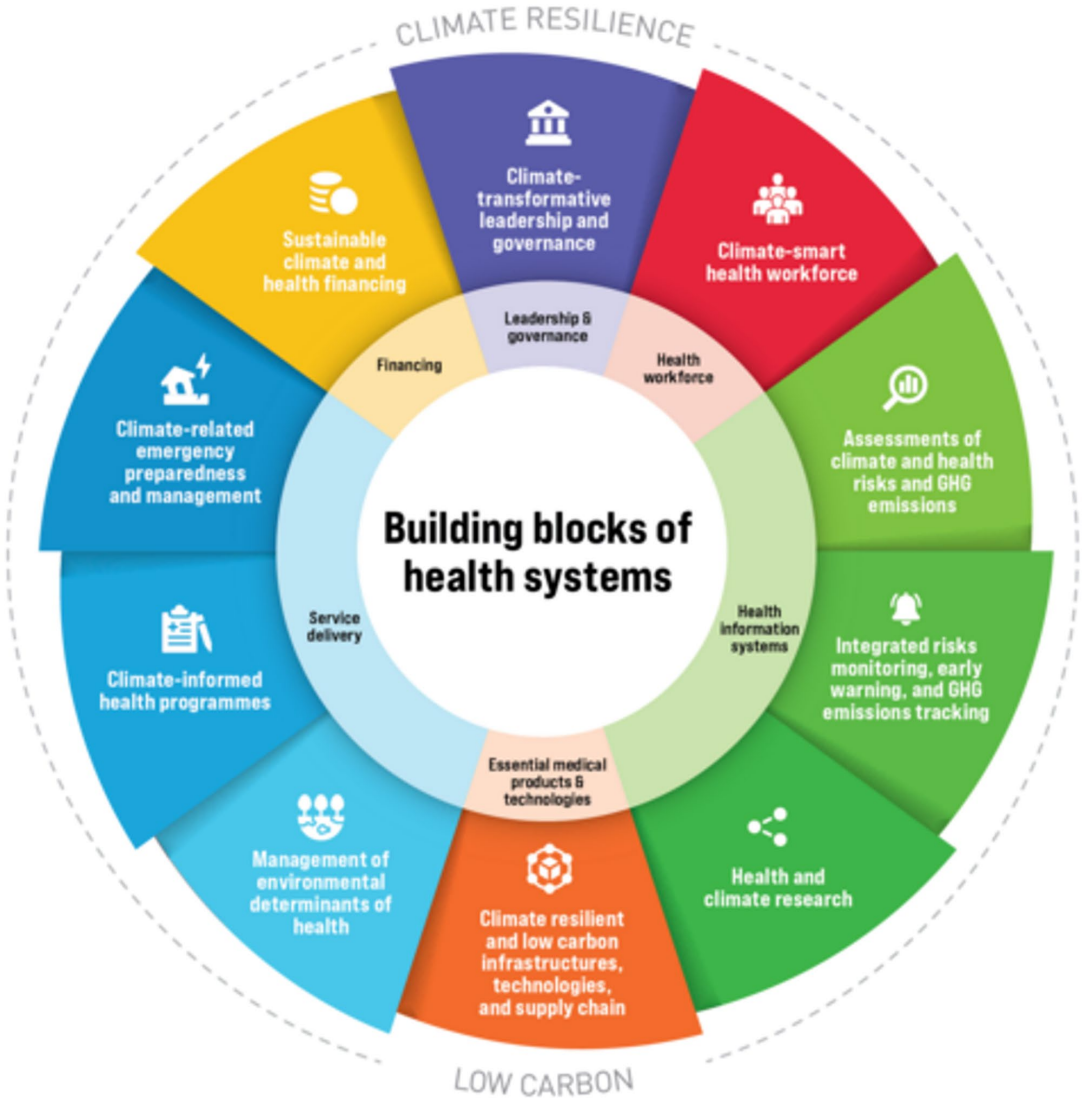
The WHO’s Operational Framework for Building Climate-Resilient and Low Carbon Health Systems. Reproduced with permission from WHO © World Health Organization, 2023

To effectively protect public health, healthcare systems must prioritize climate resilience as a core goal. The Intergovernmental Panel on Climate Change’s fourth assessment report defines climate resilience as “the ability of a social or ecological system to absorb disturbances while retaining the same basic structure and ways of functioning, the capacity of self-organization, and the capacity to adapt to stress and change” [13]. Climate resilient development integrates mitigation, adaptation, and sustainable development to promote planetary health and well-being. This is enabled through partnerships, financial commitments, and technological advancements [14]. Building climate resilience involves all actors (governments, communities, and businesses) having the capacity to anticipate climate risks and hazards, absorb shocks and stresses, and reshape and transform development pathways in the longer term [15]. The World Health Organization’s (WHO’s) (2023) Operational Framework for Building Climate-Resilient and Low Carbon Health Systems (Fig. 1) provides a comprehensive approach to strengthening healthcare system climate resilience through key interventions, including workforce development, sustainable resource management, and adaptive infrastructure. Among these interventions, the Leadership and Governance component, for example, emphasizes the importance of political leadership collaborating with different sectors to tackle climate-related health risks [16]. Similarly, the American Building Resilience Against Climate Effects (BRACE) framework emphasizes steps such as assessing vulnerabilities and implementing climate adaptation plans while integrating greenhouse gas mitigation strategies [17, 18].

According to the WHO’s 2021 Health and Climate Change Global Survey, 77% of surveyed countries reported having developed or were currently developing national healthcare and climate change plans [19]. Implementation remains limited due to barriers such as funding shortages, although key enablers include economic benefits and other facilitating factors [19–22]. Furthermore, although healthcare professionals can play a crucial role in advancing climate resilience [23, 24], significant knowledge gaps and limited awareness of climate-health linkages remain major barriers [25, 26].

Transformative leadership, effective governance, and actionable national policies are essential drivers for advancing climate resilience. These efforts should integrate health into climate agendas, promote co-benefits, enhance coordinated planning, and secure funding to develop climate-resilient systems capable of addressing diverse health threats [27–29]. Highlighting the urgency of these efforts, the Global Risks Report 2022 identified “climate action failure” as the most severe global risk over the next decade [30].

These global challenges and implementation barriers are exemplified in established healthcare systems. The UK’s National Health Service (NHS), the world’s largest publicly funded health system, exemplifies climate action by having achieved a 26% reduction in greenhouse gas emissions by 2019 compared to 1990 levels [31] and becoming the first healthcare system to embed net-zero emissions into legislation [32]. However, adaptation strategies essential for reducing climate vulnerability and strengthening resilience remain underemphasized and unfunded compared to well-supported mitigation efforts [33]. In comparison to the NHS’s ambitious net-zero commitments, Australia’s healthcare sector, with a universal public insurance scheme, operates within more modest emission reduction frameworks, targeting 43% below 2005 levels by 2030 as part of broader national climate goals. Australia has only recently introduced a National Health and Climate Strategy to complement existing state initiatives [34]. However, significant knowledge gaps remain, particularly regarding preparedness for climate-related hazards, and the overall advancement of health adaptation measures is still limited [34, 35].

The Middle East is a climate change hotspot facing significant climate risks as the region warms nearly twice as fast as the global average [36–38]. Israel’s climate-related health risks include extreme weather events, heat, air pollution, and changes in water and food availability/quality [39–41]. High temperatures and heat waves in Israel have been found to increase the risks of preterm birth [42], preeclampsia [43], stroke [44], outbreaks of West Nile fever [45], leishmaniasis [46], campylobacter among children [47], suicide [48] and snakebites [49].

Israel’s healthcare system is predominantly public, with a smaller private sector. Universal coverage is provided through national health insurance via four Health Maintenance Organizations (HMOs), ensuring equitable access to healthcare services and contributing to the nation’s overall good health outcomes [50]. However, insufficient financial resources [51] have led to shortages in personnel, infrastructure, and essential equipment. These deficiencies, along with gaps in emergency preparedness, limit the system’s ability to effectively manage both routine and emergency situations [52, 53]. Israel’s Ministry of Health plays a central role in overseeing this system, with responsibilities that include setting health priorities, allocating resources, and preparing for emergencies [54]. The Ministry’s dual role as both regulator and operator creates bureaucratic inefficiencies, conflicts of interest, and hinders long-term planning and regulation [50].

In 2018, the Israeli government formulated Resolution no. 4079 [55] stating the necessity for the preparation of a long-term National Program for Adaptation to Climate Change. However, a 2021 State Comptroller’s audit report revealed that there had been no advancement in promoting action plans and policy measures to mitigate health risks. The audit suggested that policy decisions should be made collectively by the Ministries of Health, Finance, and Environmental Protection to set targets and formulate operational strategies to enhance the Israeli healthcare system’s adaptation to climate change risks [56]. According to the State Comptroller’s extended follow-up audit (2024), the Ministry of Health made progress on four out of 13 required tasks from Resolution No. 4079. These tasks included monitoring mortalities and illness in high-risk groups, closing knowledge gaps through applied research, and preparing or updating national and local disaster preparedness plans. However, for 10 of these tasks, the required budget has still not been allocated for their implementation, and for seven of them, goals have not been set in the Ministry of Health’s work plan [57]. Additionally, Ministry of Health reports indicate other climate resilience efforts, including the taking of sporadic energy efficiency measures at various hospitals and the mapping of government hospitals through energy efficiency surveys, addressing climate-related food security issues and publishing recommendations for extreme heat situations for the elderly population [58–60].

In early 2025, the Ministry published its first official climate change document outlining preliminary adaptation and mitigation recommendations [60]. However, this plan lacks dedicated funding mechanisms and does not constitute a comprehensive national health-climate action plan aligned with WHO guidelines. A recent survey found moderate-to-high climate-related health awareness among Israeli medical staff, primarily sourced from media. The topic remains largely absent from curricula and professional training [61].

In this study we explored the barriers to and facilitators of climate resilience initiatives in Israel’s healthcare system from the perspective of decision-makers. The study’s outcomes can offer valuable insights for developing a customized framework for climate resilience in Israel’s healthcare system, informing a national health action plan on climate change and shaping future policies and strategies to strengthen healthcare systems amidst climate change.

## Methods

### Study design

We employed a qualitative exploratory design, using semi-structured interviews to collect data from officials within Israel’s healthcare system from February-September 2024.

### Participants and data collection

Participants were selected through purposive sampling, targeting key policymakers, administrators, and managers from the Ministry of Health, hospitals, and Israel’s HMOs, reflecting diverse organizations, geographic regions, and levels of responsibility across the entire Israeli healthcare ecosystem. Recruitment began with a predefined list, with participants selected on the basis of positions of influence and decision-making authority to ensure relevant expertise and valuable insights. Participants were recruited through personal inquiries via email or phone, and the study’s objectives, rationale for contacting them, and logistical arrangements were outlined. As several recruitment efforts did not result in interviews, a structured and purposive snowball sampling approach [62] was implemented, in which the researchers leveraged their professional networks to strategically expand the sample. We conducted 25 interviews (see Table 1), a sample size selected a priori drawing on empirical guidance suggesting that 9–17 in-depth interviews are typically sufficient to reach thematic saturation in studies of this scope [63]. Our ongoing transcript review confirmed saturation after 19 interviews. Twenty-two interviews were conducted face-to-face, whereas video conferencing platforms, such as Zoom, were used for three interviews. All interviews were conducted by the first author who is a graduate student with no history of employment in the healthcare system. The interviewer had no previous relationship with the interviewees, who were selected on the basis of their professional roles. The objectives of the study were introduced to participants when invited to take part in the study. Each interview lasted approximately one hour. The interviews followed an interview guide (see Appendix A) developed in accordance with the updated 2022 version of the Consolidated Framework for Implementation Research (CFIR) [64]. This framework is widely used to guide the systematic assessment of multi-level determinants of implementation and has been extensively applied within healthcare settings. The CFIR was selected to support the systematic exploration of climate resilience initiatives, barriers, and facilitators influencing these efforts, as well as participants’ views on policy development for addressing climate change impacts on health. The CFIR provides a menu of constructs across five domains. For the purposes of this study, four domains were operationalized to fit the Israeli healthcare context:

1. Outer Setting Domain: including policy environment, regulations, and incentives.
2. Inner Setting Domain: including organizational priorities and leadership engagement.
3. Individuals Domain: including beliefs and self-efficacy.
4. Implementation Process Domain: including assessing needs and tailoring strategies.

The Innovation Domain was not explicitly operationalized as the focus of the current study was on exploring the implementation environment rather than evaluating specific climate resilience interventions. Minor iterative refinements were made to the interview guide after the first two interviews to enhance clarity and ensure alignment with participants’ framing of key issues.

### Data analysis

All interviews were recorded, transcribed, and anonymized to ensure confidentiality. In the first round of coding, study team members read the transcripts, and a sample of the transcriptions was coded in ATLAS.ti software using a preliminary codebook which was developed on the basis of a comprehensive literature review, study objectives, and the CFIR Framework. In the second round of coding, the team revisited the project’s preliminary codebook for updates and coded sample transcriptions to address discrepancies. Based on the feedback from team members, the updated codebook was used to code all transcripts. The finalized codebook was then applied to all transcripts. We compared codes within and across transcripts, grouping coded extracts into sub-themes and overarching themes. An analytical framework was developed iteratively to examine the concept of climate resilience across the Israeli healthcare system. To ensure methodological transparency, we followed the Consolidated Criteria for Reporting Qualitative Research (COREQ) [65]. A completed COREQ checklist is provided in Appendix B.

**Table 1:** List of interviewees by sector and role

	Position	Organization	Region	Gender
1	Head of division - public health directorate	Ministry of health	National	Female
2	Head of division - public health directorate	Ministry of health	National	Female
3	Former head of division - medical directorate	Ministry of health	National	Female
4	Director - financial & strategic planning administration	Ministry of health	National	Female
5	Director - government medical centers directorate	Ministry of health	National	Female
6	Director - nursing division	Ministry of health	National	Female
7	Policy fellow - financial & strategic planning administration	Ministry of health	National	Female
8	District health bureaus medical director	Ministry of health	Regional	Male
9	Deputy district health bureaus medical director	Ministry of health	Regional	Male
10	Hospital director general	Hospital	North	Male
11	Hospital deputy director general	Hospital	Center	Female
12	Hospital deputy director general	Hospital	North	Male
13	Hospital administrative director	Hospital	Center	Male
14	Head of department & clinical research center director	Hospital	South	Male
15	Hospital engineer	Hospital	Center	Male
16	Head of corporate responsibility	Hospital	National	Male
17	Emergency preparedness coordinator	Hospital	South	Female
18	Emergency preparedness coordinator	Hospital	South	Female
19	Environmental health coordinator	Hospital	North	Female
20	Health services researcher	Hospital	National	Female
21	Chief physician	HMO	National	Male
22	Head of environmental sustainability	HMO	National	Female
23	District director	HMO	Regional	Male
24	Head of emergency preparedness	HMO	National	Male
25	Chair of committee, board of directors	HMO	National	Male

## Ethical considerations

The study was approved by the Institutional Human Subject Research Committee of Ben-Gurion University of the Negev (Approval #2023.380-2.380). All participants provided written consent to be interviewed and audio-recorded, after receiving an explanation of their rights and assurance that they could withdraw from the study at any time. The locations of the interviews were selected by the participants for convenience.

## Results

**Fig. 2:**
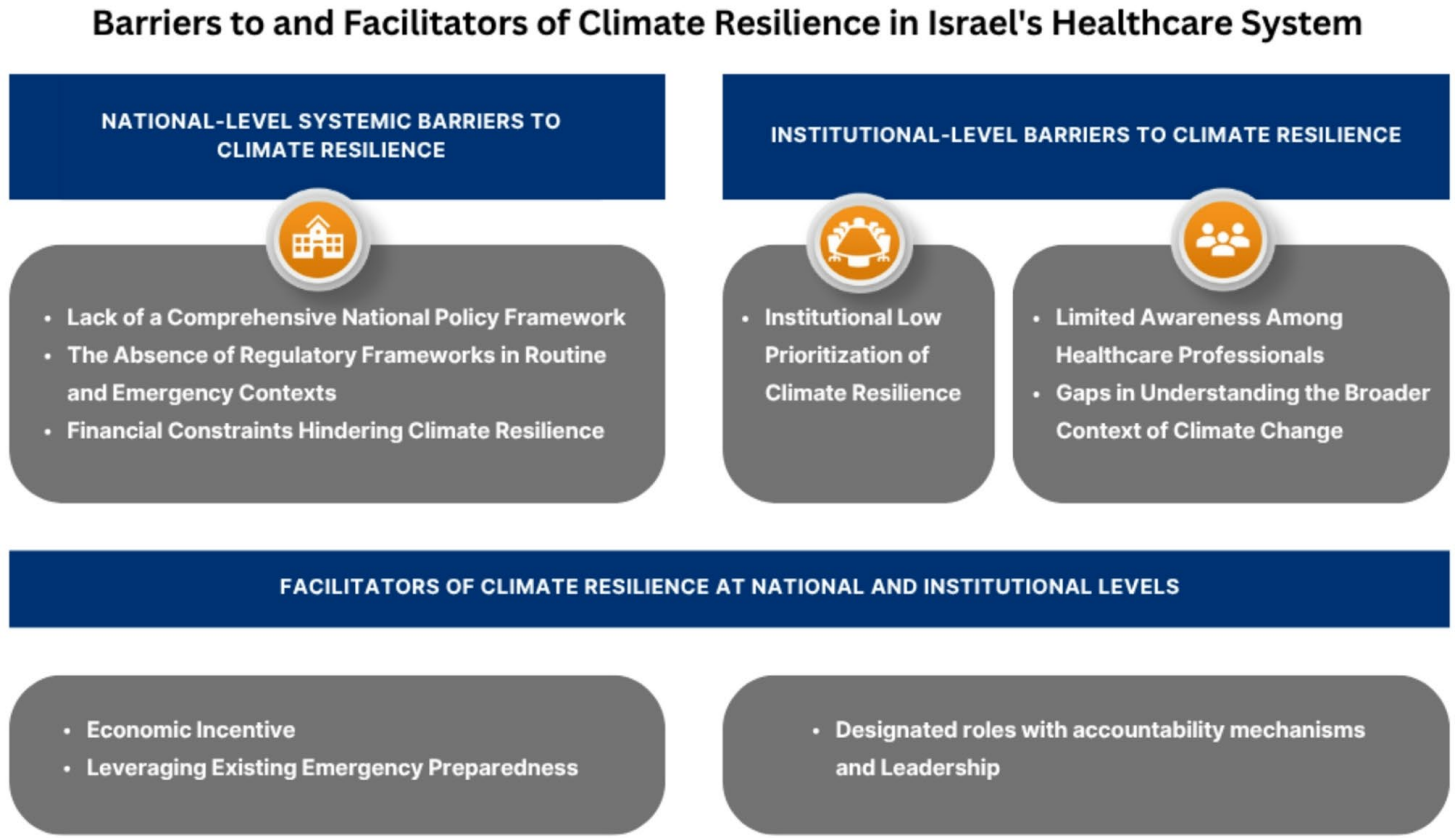
Barriers to and Facilitators of Climate Resilience in Israel’s Healthcare System

The study’s findings are structured around three central themes that emerged from the analysis, each shedding light on barriers and facilitators influencing climate resilience within Israel’s healthcare system: (1) national-level systemic barriers to climate resilience, (2) institutional prioritization and health professionals’ limited awareness as barriers to climate resilience, and (3) key facilitators supporting localized initiatives. These themes are represented in Fig. 2, which illustrates the core issues identified within each theme.

### National-level systemic barriers to climate resilience

Climate resilience within Israel’s healthcare system is significantly hindered by systemic barriers at the national level. Senior officials from hospitals, HMOs, and even the Ministry of Health consistently identified the lack of national regulation, structured guidelines, and designated funding as key challenges to effectively prepare for and respond to climate change. In the absence of national leadership, local initiatives have emerged, but their impact remains fragmented and unsustainable without coordinated governmental support. It is important to note that these interviews were conducted prior to the publication of the Ministry of Health’s climate plan in 2025.

#### Lack of a comprehensive national policy framework

The interviews highlighted the absence of a government-led, comprehensive policy framework that encompasses both adaptation and mitigation strategies – a central barrier to advancing climate resilience within Israel’s healthcare system. A Ministry of Health official from the strategic planning division explained:

*“We mapped and analyzed the current situation*,* trends*,* and the approaches of other healthcare systems around the world*,* and we realized that our system is not addressing climate change. We realized that this is the greatest crisis facing healthcare systems and public health in the 21 st century.”*

Most interviewees outside the Ministry of Health were unaware of the existence of a climate-health plan draft. An HMO board representative explained why climate change remained absent from institutional agendas:

*“Why hasn’t it come up? Because the Ministry of Health or any other government ministry has not come forward and said*,* ‘This is important.’ If someone had presented it as a measurable target—one that is monitored*,* with preparedness*,* response*,* and so on—then*,* of course*,* it would have been implemented*,* tracked*,* and included in the work plan.”*

Interviewees who actively follow climate policy developments expressed the expectation that the Ministry of Health would formulate a comprehensive policy framework. As an HMO head of environmental sustainability noted:

*"In the first draft of the Climate Law, it was mentioned that each government ministry must submit a plan. So, I’m waiting to see what the Ministry of Health will propose, how it relates to me, and what actions we can take here."*

#### The absence of regulatory frameworks in routine and emergency contexts

Participants emphasized the reliance of Israel’s healthcare system on regulation and structured protocols for operational alignment and compliance, particularly as no relevant protocols currently exist in the Israeli healthcare system to address climate change, leaving the sector without a structured framework to guide its response. A hospital official in emergency preparedness noted:

*“These [healthcare] systems work mainly through regulatory measures*,* funding*,* and established protocols. Once a requirement is in place*,* everyone aligns with it*,* just like hospitals now undergo JCI [Joint Commission International] audits.”*

Additionally, a Ministry of Health nursing division official highlighted the importance of measurable outcomes to align efforts across the sector: *“Health professionals are task-oriented; if there’s a measurable protocol*,* everyone aligns with it.”*

The interviews revealed a reliance on regulatory frameworks during emergencies (e.g., wars, earthquakes, or large-scale disasters) as examples of successful governmental coordination, but highlighted the absence of such frameworks for climate-related risks. A hospital administrative director recalled:

*“In the past*,* the Ministry of Health asked hospitals to prepare for earthquakes*,* provided funding*,* and outlined specific objectives. But no similar initiative exists for climate-related challenges.”*

An HMO executive in emergency management described the lack of coordination in receiving extreme weather updates:

*“Regarding extreme weather*,* sometimes I get updates from the Ministry of Health*,* sometimes from the meteorological service. There’s no systematic method. Sometimes*,* I find out through WhatsApp groups.”*

Another senior HMO official described their experience leading an emergency preparedness initiative, underscoring the importance of structured guidance, and the absence of such specific guidance for climate-related extreme events: “*The problem is that without practice drills or formal protocols*,* nothing progresses. There’s a need to drill these scenarios and write clear operational plans.”*

The interviews also revealed a notable gap in addressing the long-term, chronic impacts of climate change on health. As a hospital research center director stated, focusing on the here-and-now:

*“We are within the bounds of adaptation—whether physiological*,* technological*,* or behavioral. Until temperatures reach 50 degrees Celsius*,* we will likely be fine.”*

Interviewees primarily discussed acute emergencies, such as extreme weather events, with little reference to government-led policy framework for a progressively hotter climate, highlighting a critical gap in adaptation.

#### Financial constraints hindering climate resilience

Moreover, interviewees emphasized that without financial support, even the best regulations could not succeed. They repeatedly stressed the importance of dedicated funding to implement climate resilience measures effectively. An HMO district director noted:

*“If the government funds and issues guidelines*,* everyone will align with them. The more regulations there are*,* the more the Ministry of Health prioritizes this and provides support… For example*,* when they decided to make home rehabilitation a priority*,* they allocated support criteria for it*,* and the HMO adapted accordingly. That means you receive funding*,* it is designated*,* and you are also motivated to implement it.”*

Additionally, a Ministry of Health representative revealed how difficult it was to secure resources: *“For two years*,* I’ve been submitting a work plan for funding a climate and health training program but haven’t received a single shekel [local currency].”* This lack of funding limits the ability to integrate climate resilience into healthcare system planning.

According to interviewees, the absence of a clear policy framework and sufficient resources—typically tied to a national plan—forces healthcare organizations to act independently, reflecting a predominantly bottom-up approach. In this context, some leaders within hospitals, HMOs, and the Ministry of Health have initiated localized actions that prioritize climate resilience, secure resources, and integrate mitigation and adaptation strategies. Examples include improving energy efficiency, adopting renewable energy, and raising public awareness on health and climate issues. Although these efforts underscore the critical role of proactive leaders in advancing climate resilience, respondents emphasized that a top-down approach is essential to unify efforts and enable systemic, large-scale impact.

### Institutional-level barriers to climate resilience

The integration of climate resilience within Israel’s healthcare system, as identified through our analysis, is hindered by low institutional prioritization, health professionals’ limited awareness, and gaps in understanding the broader context of climate change.

#### Low institutional prioritization of climate resilience

Respondents indicated that at the institutional level, climate change was often deprioritized and overshadowed by immediate operational demands, crises, and Israel’s unique security challenges stemming from ongoing geopolitical tensions, including the October 7th events. It was frequently treated by organizations as a long-term concern, leading to its consistent marginalization in institutional planning and decision-making. An emergency preparedness coordinator remarked:

*“We have such urgent issues that we rarely think about climate threats. You could say that security concerns are a major barrier. They have a significant impact on our daily lives. Absolutely. Maybe when things are calmer*,* we’ll be able to focus on it.”*

This low institutional prioritization of climate response reflects a broader challenge within Israel’s healthcare system, which operates under budget constraints and recurrent funding reductions. These conditions foster a reactive approach to emergencies rather than enabling proactive, long-term strategic planning. As a senior official at the Ministry of Health described:

*“Climate change is on the agenda for some of us, but it’s hard to get organizational attention. There are always more pressing issues: not enough doctors, not enough staff—it’s always about what’s most urgent. And yes, the urgent overrides the important.”*

Such low prioritization can also be exacerbated by a lack of leadership support and engagement at the institutional level. Furthermore, the absence of national regulation acts as a critical barrier, undermining the ability to shape institutional prioritization efforts for advancing climate resilience. As a senior public health official from the Ministry of Health remarked:

*“Climate change policy is still seen as ‘nice to have.’ People understand it’s important*,* but they don’t grasp the urgency. In my opinion*,* neither the CEO nor the deputy CEO [see the urgency]… Climate change policy is perceived as a luxury*,* something for someone else to handle. We’re focused on saving lives.”*

#### Limited awareness among healthcare professionals

Healthcare professionals’ perceptions further compound these institutional challenges. Across the interviews, there was a widespread lack of awareness regarding the urgency and severity of climate change’s risks to public health, accompanied by a generally low understanding of the connection between climate and health. Many perceived climate change as irrelevant to their roles. As a senior hospital manager stated: *“If you talk to a hospital manager about climate change*,* they’ll ask*,* ‘Why is this relevant to me?’”* Another respondent highlighted a similar sentiment: *“The increase in heatwaves isn’t seen as requiring special preparation because Israel is well prepared for heat.”*

These findings highlight the critical interplay between institutional dynamics and professionals’ perceptions. The systemic lack of prioritization creates an environment where healthcare professionals view climate resilience as external to their roles.

#### Gaps in understanding the broader context of climate change

Although assessing knowledge was not the primary focus of this research, the results revealed a pervasive gap in participants’ understanding of the broader climate change context and its specific health-related consequences, particularly among those without direct climate-related responsibilities. A hospital deputy director summarized this lack of knowledge:

*“Most hospital directors and management teams don’t have much knowledge [about climate change]. The little I know comes from roles I’ve held in the past.”*

Some participants also disclosed that their knowledge about climate change and health was acquired informally, primarily through the media, rather than through professional education or training. A senior HMO officer stated: *“I know very little… what I hear on TV or read in the newspaper. It’s not a topic that’s ever been prioritized or something I’ve taken responsibility for.”* Even a hospital emergency preparedness official said: *“At conferences or meetings*,* there’s never been a session on climate change as a health threat. I haven’t encountered it in the field of emergency preparedness.”*

Interestingly, the interviews prompted a shift in participants’ awareness. As a senior HMO officer noted: *“Now I understand how important this is; I’ll bring it up at the next management meeting.”*

Although climate change poses significant health risks, it is often viewed as a secondary concern, influenced by healthcare professionals’ limited awareness. These factors contribute to a cycle of inaction, delaying the integration of climate resilience measures.

### Facilitators of climate resilience at national and institutional levels

We identified several facilitating factors that supported local initiatives (i.e., mitigation, adaptation, or a combination of the two) and contributed to institutions’ climate resilience. According to interviewees, when effectively implemented, these factors create a set of actions that allow healthcare institutions to practically address the challenges posed by climate change. The facilitating factors comprised economic incentives, leveraging existing emergency preparedness frameworks to dedicated roles, and oversight mechanisms.

#### Economic incentives

Economic factors were frequently cited as powerful facilitators for promoting climate resilience. Although energy-saving projects, such as upgrading equipment, installing solar panels, or implementing automated energy-efficient systems, are often undertaken with the primary goal of reducing operational costs, these projects also contribute to environmental sustainability. An HMO medical director explained:

*“Several years ago, we implemented smart energy systems in all our buildings. Would I say they did this because of climate change? I don’t think so. Someone calculated that it would lower the electric bill.”*

Despite the need for upfront investment, interviewees emphasized the importance of a clear return on investment (ROI) to justify expenditures on climate resilience initiatives. One hospital administrator explained: *“In cases where investments are required*,* there are general guidelines we follow—if the ROI is expected within five years*,* such initiatives are typically approved.”* This pragmatic approach underscores how aligning sustainability efforts with financial viability can help overcome budgetary constraints.

There are additional advantages that also encourage initiatives promoting climate resilience. As a hospital administrative director stated about an innovative energy storage project: *“It’s green*,* it saves money*,* it’s innovative*,* and it positions us well globally. Who wouldn’t take on a project like that?”* This perspective illustrates how sustainability efforts, when framed as not only cost-saving but also forward-thinking and globally competitive, can further motivate healthcare institutions to embrace climate resilience measures.

#### Leveraging existing emergency preparedness

According to interviewees, Israel’s established frameworks for managing conflict-related emergencies and earthquake preparation present a unique opportunity for enhancing climate resilience. Currently, Israel’s emergency preparedness focuses heavily on the “blackout scenario,” referring to widespread power outages caused by missile strikes during the Swords of Iron War, though similar disruptions could also result from extreme weather events. Many aspects of emergency preparedness, such as contingency planning for power outages or managing vulnerable populations’ needs, can be applied directly to climate-related emergencies. As one senior HMO executive explained:

*“Today*,* all of our unified clinics* [primary care clinics providing services to patients of all HMOs in emergency situations] *have generators and backup systems*,* including solar installations in some cases.”*

Another senior official at an HMO explained: *“In the new buildings… there are plans to install solar systems… I don’t think our logistics manager is really an environmental advocate. He’s simply looking to… ensure we have an automatic system for power outages instead of relying on generators.”*

Adapting and expanding the objectives of existing emergency preparedness strategies offers a cost-effective way to strengthen climate resilience, addressing not only infrastructure but also procedures and mechanisms. As an HMO executive described:

*“The Ministry of Health created a mechanism* [during the recent war] *where we receive notifications about power outages by region*,* including their expected duration. This enables us to prepare and even plan patient evacuations if necessary.”*

Leveraging Israel’s existing emergency preparedness frameworks—originally designed for conflict-related crises and power outages—presents a strategic and cost-effective opportunity to enhance climate resilience.

#### Designated roles with accountability mechanisms and leadership

One facilitator identified by participants was the establishment of a dedicated climate resilience role within healthcare organizations. Interviewees emphasized the importance of designating someone who would be responsible for managing climate resilience to ensure that climate-related concerns were prioritized and that ongoing oversight and accountability were maintained. As one senior hospital executive noted: *“The way it works is that you appoint someone responsible*,* give them specific tasks*,* and they conduct oversight and report on it—this is how things start moving.”*

As part of her dedicated role in advancing environmental and climate-related initiatives, and given her expertise in the field, the HMO head of environmental sustainability stated: *“What matters to me is to get things moving… Because of my broad familiarity with the processes… I knew exactly who to contact to make sure this got to the right person.”*

Interviewees highlighted that effective climate resilience initiatives require individuals in these roles to demonstrate leadership skills, as strengthening climate resilience often necessitates driving systemic changes. Interviewees with notable leadership abilities described how they successfully spearheaded initiatives that positively impacted the healthcare system’s climate resilience. As a hospital deputy director general testified:

*“Each deputy is assigned specific areas of responsibility. My role focuses on innovation*,* which incorporates climate-related issues. Many of our climate and innovation initiatives are pioneering in Israel and*,* in some cases*,* globally. The hospital engineer leads these efforts*,* as he possesses specialized expertise in this area.”*

These findings demonstrate that integrating financial, operational, and leadership strategies is essential for effectively enhancing climate resilience in healthcare systems. Moreover, these facilitating factors can act as catalysts for broader organizational initiatives, highlighting their critical role in driving sustainable and systemic change.

## Discussion

The aim of this study was to analyze the barriers to and facilitators of climate resilience initiatives in Israel’s healthcare system from the perspective of policymakers, administrators, and healthcare managers. The findings highlight systemic barriers, including healthcare professionals’ limited awareness and institutional prioritization, as well as facilitating factors. In the following discussion we situate these findings within the broader challenges facing Israel’s healthcare system, as well as the global complexities of policy implementation in climate resilience and health adaptation, and offer policy recommendations for advancing climate resilience in Israel’s healthcare system.

### Low priority of climate issues in israel’s healthcare system

This study underscores the low prioritization of climate issues within Israel’s healthcare system, where climate change is frequently perceived as a low priority. This low prioritization can be understood through institutional theory, which posits that organizations tend to conform to prevailing norms and peer behaviors to maintain legitimacy. Such institutional isomorphism (where organizations mimic counterparts facing similar constraints) often results in path dependency, limiting responsiveness to emerging challenges such as climate change, and particularly under conditions of uncertainty and limited resources [66].

Additionally, climate-related concerns are outweighed by immediate operational challenges and Israel’s unique geopolitical and security considerations. This prioritization is evident in the government’s budget, where significant allocations to national security concerns come at the expense of healthcare investment [67]. The chronic underfunding of healthcare infrastructure, compounded by insufficient dedicated resources for climate resilience, reflects global financing barriers to advancing climate adaptation in healthcare systems [19, 20, 22]. Notably, only 0.5% of multilateral climate finance globally is allocated to health projects, despite evidence that climate-resilient health interventions can significantly mitigate the health impacts of climate change [68].

Although certain initiatives within Israel’s healthcare system contribute to climate resilience, their primary goal is not climate-related; instead, they frame cost-saving measures (e.g., in terms of energy efficiency) as a key facilitator for driving climate action, consistent with global findings [21]. Similar to previous studies from other regions in the world [25, 26], this study highlights a significant gap in healthcare professionals’ awareness and knowledge. Israeli healthcare professionals demonstrate limited awareness of climate change severity and urgency despite global recognition of its public health significance, undermining their capacity to motivate resilience measures and guide climate-related health interventions [69–71]. Understanding risk perception patterns among Israeli healthcare professionals provides additional insight into the observed low prioritization of climate issues. Research on healthcare workers’ decision-making during COVID-19 demonstrates that risk perception in Israel’s healthcare system is significantly influenced by immediate threat salience and exhibits temporal decline during ongoing crises [72]. This pattern may partially explain why long-term, gradually evolving threats such as climate change struggle to maintain attention in Israel’s healthcare system.

Although some interviewees demonstrated a growing interest in climate resilience during the interviews and recognized the urgent need for action, these shifts in awareness, though promising, are not sufficient without sustainable, system-wide action. Moreover, effective adaptation requires not only informed professionals but also strong political will to prioritize and address these challenges [73].

### Integrating climate resilience into emergency preparedness

The study findings point to a notable gap in climate preparedness within Israel’s healthcare system. Although participants often showed limited awareness of the broader threat posed by climate change and the necessity of preparedness, they acknowledged, when prompted, that addressing such risks is important and would require substantial effort. Such efforts include securing dedicated funding, developing comprehensive standard operating procedures, establishing collaborative frameworks, and conducting regular exercises and drills—similar to those implemented for other types of emergencies managed by the healthcare system [74–76].

The interviews further suggest that preparedness for security-related crises significantly contributes to readiness for climate-related emergencies, as protocols and infrastructures often overlap. This intersection reflects the broader climate-conflict nexus [77], which in low- and middle-income countries is often framed as climate change acting as a stressor that increases the risk of conflict. However, in high-income countries like Israel, with its multi-crisis context, the nexus is conceptualized through preparedness efforts, where integrated planning can address both climate-related and security threats, ultimately strengthening overall resilience.

The heightened awareness of security emergency preparedness in Israel, driven by ongoing security challenges, particularly following the war that started on October 7th 2023, alongside ongoing efforts for earthquake preparedness, provides a foundation for addressing these gaps through the adoption of an All-Hazards Approach [78, 79]. This approach, which has demonstrated effectiveness in managing security-related emergencies and earthquake preparedness in Israeli hospitals, is a comprehensive emergency preparedness framework that considers the full scope of emergencies or disasters when planning for response capacities and risk mitigation efforts. Consistent with both our interview findings and the existing literature, resource shortages represent a critical barrier to addressing climate-related threats effectively [19–22]. Leveraging existing resources and minimizing duplication can result in a cost-effective strategy for integrating climate-related risks without the need for entirely new structures (74, 79). Addressing risk and resilience together is crucial, as their inherent interconnection enables a more integrated and effective approach to tackling complex challenges by leveraging synergies and ensuring balanced responses [80]. For instance, the “blackout scenario,” and the need to reinforce power infrastructures against missile-related attacks, could also apply to outages caused by climate-related extreme weather events; the diversification of energy sources and strengthening of energy security through the development of energy storage systems could improve readiness for both security-related and climate-related emergencies. However, critics of the All-Hazards Approach argue that its inherent weaknesses (e.g., its broad focus) may lead to insufficient preparation for specific, high-priority threats, thereby hindering effective disaster planning and response. They advocate for a Top-Hazards Approach that prioritizes resources based on local risk indicators and focuses on the highest-risk hazard [79, 81]. Both the All-Hazards Approach and the Top-Hazards Approach offer viable pathways for strengthening climate resilience.

Whereas acute climate events received attention in the interviews, long-term challenges were largely overlooked. This oversight contrasts with the WHO’s Quality Criteria for Health National Adaptation Plans [28], which advocate for comprehensive, integrated approaches that address both immediate and long-term climate risks. Climate preparedness must be viewed as a dynamic, ongoing process, supported by a structured methodology to assess and improve readiness for evolving risks [82, 83].

### Policy recommendations for advancing climate resilience

Given the escalating impacts of climate change and the significant health risks they pose, the absence of a comprehensive national policy framework in Israel’s healthcare system (including dedicated funding and binding regulations) has severe consequences and undermines the system’s ability to address current and future challenges effectively. Interviewees highlighted the lack of national leadership, coordination, regulatory clarity, and financial support, which severely limit the integration of climate resilience into healthcare system operations. Although Israel’s Ministry of Health recently released an official climate change document, its impact remains uncertain. Interviewees, whose perspectives were gathered before its release, were unaware of its draft version. The absence of a clear dissemination strategy, a designated role to oversee its execution, and dedicated funding raise concerns about its effective implementation.

Examples from other countries such as France’s National Heat Wave Plans demonstrate how a well-structured, government-led climate health policy can save lives. Real-time health data surveillance, targeted interventions, and an advance warning system effectively reduced excess deaths in France during the 2006 heat wave (vs. the catastrophic 2003 event), underscoring the critical importance of comprehensive climate resilience strategies in safeguarding public health [84].

Legislation and national programs are critical for driving quality improvements in Israel’s healthcare system [71, 85], and mandatory policy measures supported by robust reporting and monitoring are more effective than voluntary agreements in achieving systemic improvements [86]. Our findings underscore that a key enabler for advancing climate resilience is the establishment of designated roles (i.e., climate resilience officer/coordinator) with clear accountability mechanisms. This study, aligning with global research, highlights the importance of proactive, knowledgeable leaders in advancing climate action [87, 88] particularly in the absence of a comprehensive national policy [89]. Additionally, according to Hartwell et al. [20], institutional dynamics, including implementation within the institution, staff capacity to engage effectively, and the prioritization of climate issues, can either facilitate or hinder climate-related initiatives. Integrating climate resilience training into professional development is essential for equipping leaders with the tools necessary to drive systemic, sustainable change [90].

Furthermore, this study’s findings align with the broader global understanding of the need for systemic approaches to climate resilience in healthcare systems [29]. The health impacts of climate change necessitate urgent involvement across all levels of Israel’s healthcare system, through a coordinated, government-led framework that includes funding, education, and comprehensive preparedness to strengthen climate resilience. Such a framework should leverage local/institutional leadership, economic incentives, and existing emergency response infrastructures to address knowledge gaps and systemic barriers, aligning with “gold standard” global frameworks such as the WHO’s operational framework for building climate-resilient health systems [16, 91–93].

In light of the study’s findings, in Table 2 we present policy recommendations for strengthening the resilience of Israel’s healthcare system.

**Table 2:** Key policy recommendations

Key policy recommendations	Description
Establish a Unified, Government-Led Framework	Develop a national policy framework integrating adaptation and mitigation strategies within the healthcare system. Include clear, measurable outcomes and standardized protocols to ensure coordination and accountability.
Allocate Dedicated Funding for Climate Resilience	Secure sustained government-led funding to support infrastructure upgrades, public awareness campaigns, and professional training. Prioritize equitable distribution to support vulnerable populations and resource-limited regions.
Appoint Professional Personnel in Key Leadership Roles	Designate skilled professionals to lead climate-health initiatives at national and institutional levels. Focus on strategy development, inter-agency coordination, and evidence-based implementation.
Strengthen Local Leadership within a Unified Framework	Equip local healthcare leaders with the tools, resources, and regulatory support necessary to scale initiatives effectively. Align these local efforts with national guidelines for a cohesive system.
Integrate Emergency Preparedness Under One Framework	Consolidate climate-related emergency preparedness (e.g., heatwaves, floods) with other crises like earthquakes and security events under a single comprehensive framework to optimize resources and streamline responses.
Promote Research and Evidence-Based Solutions	Invest in interdisciplinary research to understand the health impacts of climate change and develop innovative adaptation and mitigation strategies. Highlight co-benefits such as improved public health and cost savings.

### Limitations

This study’s qualitative design prioritized exploring systemic barriers to and facilitators of climate resilience rather than directly evaluating the implementation or outcomes of specific interventions. Additionally, reliance on self-reported data from interviews introduces the potential for response bias, as participants may have emphasized or minimized certain issues based on their individual experiences or roles. Moreover, we interviewed specific individuals in particular positions, potentially not providing a comprehensive representation of all relevant stakeholders. Member checking was not conducted due to participants’ senior roles and limited availability, which may limit opportunities for validation of the findings from participants’ perspectives. Finally, the focus on Israel’s healthcare system may limit the applicability of the findings to other geopolitical or cultural contexts, reducing their generalizability.

Importantly, the study was conducted during a period of war, when much of the healthcare system’s attention was diverted, potentially influencing participants’ responses and the overall data collected.

## Conclusions

This study provides a comprehensive analysis of climate resilience within Israel’s healthcare system, examining initiatives designed to enhance resilience and identifying barriers and facilitators influencing these efforts. The findings support the development of effective policies by leveraging existing mechanisms, such as integrating climate resilience into emergency preparedness frameworks, while addressing local challenges such as the low prioritization of climate issues and limited awareness.

This research is particularly timely in light of Israel’s advancement of the Climate Bill in its first reading [94]. It provides a unique opportunity for the Ministry of Health to design a comprehensive national health and climate plan that tackles identified barriers and leverages enabling factors to strengthen the system’s climate resilience. Without such a framework, local initiatives and solutions, although valuable, will remain limited in scope and impact.

## Supplementary Information

Supplementary Material 1.

Supplementary Material 2.

## Data Availability

Data sharing is not applicable to this study to protect participant confidentiality and address privacy concerns.
