# Supplementary material for: Toward climate resilience in Israel’s healthcare system: decision-makers' perspectives
Source: Isr J Health Policy Res. 2025 Nov 24;14:68. doi: 10.1186/s13584-025-00729-w (PMC12642036; doi:10.1186/s13584-025-00729-w)
Supplement: Supplementary file 1 — Supplementary Material 1. [file 13584_2025_729_MOESM1_ESM.docx]

**Appendix A: Interview Guide**

# Introduction

Thank you for agreeing to participate in this interview. My name is ______. I am conducting research on climate resilience in health systems—the ability of health systems to ensure the continuous provision of safe and high-quality care to the public amidst the uncertainties and complexities created by climate change. According to the WHO framework, climate-resilient health systems can anticipate, respond to, and adapt to extreme climate events.

This study aims to identify barriers to and facilitators of climate resilience initiatives within Israel's healthcare system. Its findings can provide valuable insights to inform future policies and strategies aimed at enhancing climate resilience in Israel's healthcare system.

As described in the informed consent form provided to you earlier, participating in this interview is voluntary. You can choose not to answer a question or you can stop the interview at any time.

With your permission, I would like to audio-record the interview. The audio-recording and transcripts will be stored on a secure server. Your personal details will remain private, and the content of the interview will only be used for research purposes. If, at any time, you feel that the questions are too sensitive, I can pause the recording during that part of the interview.

Do you have any questions?

Ok, I will start the recorder.

# Interview Questions

1. Could you please describe your role and responsibilities in your organization?
2. Could you share, from your perspective, what are the main public health impacts of climate change?

(Brief explanation of the WHO model for climate resilience in health systems)

1. What do you think about the WHO model?
   1. To what extent does this framework align with operational realities in your organization?
2. How, if at all, does climate resilience intersect with your professional responsibilities?
   1. If a decision were made to prepare and implement a program to develop climate resilience in your organization, how would you feel about that?
   2. What do you think should be the main tasks in such a program?
3. Have any actions been taken so far to develop climate resilience in the organization you work in?
   1. What actions have been taken?
   2. Why were these actions taken?
   3. Who led these actions?
   4. What factors enabled these actions?
   5. What were the barriers?
4. How is climate resilience policy reflected in your organization?
   1. What is your organization’s mandate regarding climate change?
   2. How are decisions made regarding climate change?
   3. What sources of information are used in shaping such policies?
   4. Is there collaboration or learning with/from other organizations in Israel or abroad?
   5. To what extent would a climate resilience program be an advantage for your organization compared to other organizations in Israel and worldwide?

(Consider factors such as: structural characteristics, organizational culture, organizational climate for innovation, available resources, political conditions, technological availability, environmental conditions)

1. Are you aware of any future activities or policy development processes regarding climate resilience in your organization? What are they?
2. What do you think is the role of the health system in Israel in relation to climate change?
   1. Do you think the health system has a role in addressing the impacts of climate change?
   2. Is the health system currently offering solutions to the challenges posed by climate change?
   3. What steps could the health system take to strengthen its climate resilience?
3. The Ministry of Health is in the process of developing a preparedness plan for the health system to address climate change. What do you think should be considered to ensure the effective implementation of a climate resilience program in the Israeli health system?
   1. How ready is the health system to implement such a program?
   2. What will be the main challenges in implementing the program?
   3. Do you think these challenges can be overcome?
   4. What factors might enable its implementation?
   5. What might be the barriers?
4. Do you think there is a connection between the preparedness of your organization, specifically, and the health system, in general, for emergency events* and preparedness for the impacts of climate change?
   1. Could you explain?
   2. How, if at all, might existing emergency preparedness for scenarios such as extended power outages (damage to power infrastructure causing prolonged electricity shortages) contribute to preparedness for addressing climate change impacts?

(According to the National Emergency Management Authority (NEMA), emergency situations may arise from one of the following: limited conflict or war; conventional or unconventional terrorism, including disruption of information systems leading to risk to life or damage to infrastructure; natural disasters such as earthquakes and tsunamis; epidemics; operational failures causing hazardous material or radiological substance spread that could lead to a mass disaster.)

1. What key events, in your opinion, would make climate resilience a priority in the health system?

Thank you very much for your cooperation and for the informative and enriching conversation. Would you like to add anything else?
