# Supplementary material for: Toward climate resilience in Israel’s healthcare system: decision-makers' perspectives
Source: Isr J Health Policy Res. 2025 Nov 24;14:68. doi: 10.1186/s13584-025-00729-w (PMC12642036; doi:10.1186/s13584-025-00729-w)
Supplement: Supplementary file 2 — Supplementary Material 2. [file 13584_2025_729_MOESM2_ESM.docx]

**Appendix B: Consolidated Criteria for Reporting Qualitative Studies (COREQ): 32-item checklist**

| **No.** | **Item** | **Guide questions/description** | | **Page** |
| --- | --- | --- | --- | --- |
| **Domain 1: Research team and reflexivity**  ***Personal characteristics*** | | | | |
| 1. | Interviewer/ facilitator | Which author/s conducted the interviews or focus group? | **IL** | Title page |
| 2. | Credentials | What were the researcher’s credentials? e.g., PhD, MD | **IL** holds an MA and conducted the study as part of her preliminary doctoral research. **SS**, **MN**, and **AR** hold PhDs and contributed to the study design, supervision, and analysis. | Not in manuscript |
| 3. | Occupation | What was their occupation at the time of the study? | At the time of the study, **IL** was a graduate student. **SS**, **MN** and **AR** were academic faculty members, all based in schools of public health in Israel. | Not in manuscript |
| 4. | Gender | Was the researcher male or female? | All female. | Not in manuscript |
| 5. | Experience and training | What experience or training did the researcher have? | **IL** received training in qualitative research methods during her graduate studies and was closely supervised throughout the study. **SS**, **MN**, and **AR** are experienced qualitative researchers with multiple peer-reviewed publications using qualitative methodologies in public health and healthcare systems research. | Not in manuscript |
| ***Relationship with participants*** | | | | |
| 6. | Relationship established | Was a relationship established prior to study commencement? | No. | 9 |
| 7. | Participant knowledge of the interviewer | What did the participants know about the researcher? e.g., personal goals, reasons for doing the research | Participants were informed that the interview was part of a research study on climate resilience in the Israeli healthcare system. They were informed that the study was funded by the National Institute for Health Policy Research and was supervised by **Prof. Maya Negev** (University of Haifa) and **Dr. Stav Shapira** (Ben-Gurion University). They were informed that the aim of the study was to identify strategies and practices related to health and climate adopted by actors within the healthcare system, and to analyze barriers and facilitators. | 8 |
| 8. | Interviewer characteristics | What characteristics were reported about the interviewer/facilitator? e.g., bias, assumptions, reasons and interests in the research topic | The objectives of the study were introduced to participants when invited to take part in the study. | 9 |
| **Domain 2: Study design**  ***Theoretical framework*** | | | | |
| 9. | Methodological orientation and theory | What methodological orientation was stated to underpin the study? e.g., grounded theory, discourse analysis, phenomenology, content analysis | A qualitative exploratory design, using semi-structured interviews. The study was guided by the Consolidated Framework for Implementation Research (CFIR), a comprehensive framework designed to assess contextual determinants of implementing health interventions and innovations. | 8-10 |
| ***Participant selection*** | | | | |
| 10. | Sampling | How were participants selected? e.g., purposive, convenience, consecutive, snowball | Purposive sampling was used to select key policymakers, administrators, and managers from the Ministry of Health, hospitals, and HMOs across Israel. Snowball sampling was employed to strategically expand the sample through professional networks. | 8-9 |
| 11. | Method of approach | How were participants approached? e.g., face-to-face, telephone, mail, email | Participants were approached through personalized emails and phone calls. | 8 |
| 12. | Sample size | How many participants were in the study? | Twenty-five interviews were conducted. | 9 |
| 13. | Non-participation | How many people refused to participate or dropped out? Reasons? | Five individuals who were approached declined or did not respond. No participants dropped out of the study. | Not in manuscript |
| ***Setting*** | | | | |
| 14. | Setting of data collection | Where was the data collected? e.g., home, clinic, workplace | In-person interviews (22) were conducted at the participants’ workplaces. Three interviews were conducted remotely via Zoom. | 9 |
| 15. | Presence of non-participants | Was anyone else present besides the participants and researchers? | In two of the interviews, the interviewees invited administrative staffers to participate in the interview. | Not in manuscript |
| 16. | Description of the sample | What are the important characteristics of the sample? e.g., demographic data, date | Participants were key policymakers, administrators, and managers from the Ministry of Health, hospitals, and Israel's HMOs, reflecting diverse organizations, geographic regions, and levels of responsibility across the entire Israeli healthcare ecosystem. Table 1 provides an overview. | 8&10-12 |
| ***Data collection*** | | | | |
| 17 | Interview guide | Were questions, prompts, guides provided by the authors? Was it pilot-tested? | A semi-structured interview guide with predefined questions and prompts was developed by the authors. The guide was not formally pilot-tested, but it was reviewed by the research team and refined prior to data collection (Appendix A). | 9 |
| 18. | Repeat interviews | Were repeat interviews carried out? If yes, how many? | No. | NA |
| 19. | Audio / visual recording | Did the research use audio or visual recording to collect the data? | Audio recording. | 10 |
| 20. | Field notes | Were field notes made during and/or after the interviews? | Yes. | Not in manuscript |
| 21. | Duration | What was the duration of the interviews of focus groups? | Interviews lasted approximately one hour. | 9 |
| 22. | Data saturation | Was data saturation discussed? | The research team monitored emerging themes throughout data collection and determined that thematic saturation was reached when no new themes emerged after the 19th interview. | 9 |
| 23. | Transcripts returned | Were transcripts returned to participants for comments and/or corrections? | No. | NA |
| **Domain 3: Analysis and findings**  ***Data analysis*** | | | |  |
| 24. | Number of data coders | How many data coders coded the data? | One (**IL**). The team reviewed the coding process. | 10 |
| 25. | Description of the coding tree | Did authors provide a description of the coding tree? | No. | NA |
| 26. | Derivation of themes | Were themes identified in advance or derived from the data? | A preliminary codebook was developed on the basis of the CFIR framework, literature review, and study objectives. Additional codes and sub-themes were added inductively as new insights emerged during data analysis. | 10 |
| 27. | Software | What software, if applicable, was used to manage the data? | ATLAS.ti software was used to manage, code, and analyze the interview transcripts. | 10 |
| 28. | Participant checking | Did participants provide feedback on the findings? | No | NA |
| ***Reporting*** | |  | |  |
| 29. | Quotations presented | Were participant quotations presented to illustrate the themes/findings?  Was each quotation identified? e.g., participant number | Yes. | 12-24 |
| 30. | Data and findings consistent | Was there consistency between the data presented and the findings? | Yes. | 12-24 |
| 31. | Clarity of major themes | Were major themes clearly presented in the findings? | Three key themes were identified in the study and presented in the manuscript. All of the themes were discussed in the Results section. | 12-24 |
| 32. | Clarity of minor themes | Is there a description of diverse cases or discussion of minor themes? | In addition to the main findings, relevant sub-themes and less dominant patterns were described to provide depth and nuance. | 12-24 |

Reference:

Tong, A., Sainsbury, P., Craig, J. (2007) Consolidated criteria for reporting qualitative research (COREQ): a 32-item checklist for interviews and focus groups. International Journal Quality Health Care 19(6):349–357
